# Circulating levels and subcutaneous adipose tissue gene expression of pigment epithelium-derived factor in polycystic ovary syndrome and normal women: a case control study

**DOI:** 10.1186/1477-7827-11-77

**Published:** 2013-08-14

**Authors:** Sheila B Lecke, Debora Morsch, Poli M Spritzer

**Affiliations:** 1 Division of Endocrinology, Gynecological Endocrinology Unit, Hospital de Clínicas de Porto Alegre, Porto Alegre, Brazil; 2 Universidade Federal de Ciências da Saúde de Porto Alegre, Porto Alegre, Brazil; 3 Department of Physiology, Laboratory of Molecular Endocrinology, Universidade Federal do Rio Grande do Sul, Porto Alegre, Brazil

**Keywords:** Insulin resistance, Pigment epithelium-derived factor, Obesity, Polycystic ovary syndrome, mRNA

## Abstract

**Background:**

Polycystic ovary syndrome (PCOS) has been recognized as a metabolic disorder, manifested by abdominal obesity, insulin resistance, dyslipidemia and hypertension. Pigment epithelium-derived factor (PEDF), a member of the serine protease inhibitor family, is a pleiotropic protein known for its antiangiogenic, antioxidant, and neuroprotective properties and has been shown to induce insulin resistance and play a role in glucose metabolism. Recent studies investigating circulating PEDF levels show elevated serum PEDF in association with insulin resistance in normal-weight women with PCOS, but not in obese PCOS patients. The aims of this study were 1) to assess PEDF gene expression in subcutaneous adipose tissue (scAT) from women with PCOS and nonhirsute, ovulatory controls, and 2) to determine the circulating levels of PEDF in these groups.

**Methods:**

Total RNA was extracted from adipose tissue biopsy samples and reverse-transcribed to cDNA. Real-time quantitative PCR was performed to determine relative gene expression levels.

**Results:**

The 22 women with PCOS and 14 non-PCOS controls included in the study had similar age, BMI, and fasting glucose, triglycerides, and HDL-cholesterol levels. Participants with PCOS exhibited higher 2 h oral glucose tolerance test levels (p = 0.006), total (p = 0.026) and LDL-cholesterol (p = 0.036), Ferriman-Gallwey score (p = 0.003) and total testosterone (p = 0.001) as compared to controls. BMI-adjusted PEDF serum levels and scAT gene expression were similar in the PCOS and control groups (p = 0.622 and p = 0.509, respectively). Circulating PEDF levels were not associated with scAT PEDF gene expression. Multiple regression analysis revealed that, in women with PCOS, insulin contributed positively and significantly to serum PEDF (p = 0.027), independently of testosterone.

**Conclusion:**

Serum PEDF levels and scAT gene expression were associated with metabolic risk factors, but did not differ between women with PCOS and age- and BMI-matched controls. Circulating levels and scAT gene expression of PEDF were not associated in the study subjects, suggesting additional sources for PEDF in addition to or instead of fat tissue.

## Background

Polycystic ovary syndrome (PCOS), the most prevalent endocrine disorder in women of reproductive age, affects 9 to 18% of women worldwide, depending on diagnostic criteria [1]. PCOS has also been recognized as a metabolic disorder, manifested by abdominal obesity, insulin resistance, dyslipidemia and hypertension. These factors seem to increase the risk of cardiovascular disease and metabolic syndrome in this population [2-8]. Obesity has a pooled estimated prevalence of 49% among women with PCOS, as shown by a recent meta-analysis [9].

Pigment epithelium-derived factor (PEDF), a member of the serine protease inhibitor family, is a pleiotropic protein known for its antiangiogenic, antioxidant, and neuroprotective properties [10,11]. Cumulating evidence suggests that PEDF is associated with adiposity, type 2 diabetes, and the metabolic syndrome [12,13]. Furthermore, PEDF has been shown to be an adipokine (adipocyte-secreted protein) that induces insulin resistance and plays a role in glucose metabolism [14-18].

Recent studies on circulating PEDF levels have shown elevated serum PEDF in association with insulin resistance in normal-weight women with PCOS [19], but not in obese PCOS patients [20]. Therefore, our aim was to characterize PEDF gene expression in subcutaneous adipose tissue (scAT) from women with PCOS and nonhirsute, ovulatory control women, and to determine circulating levels of PEDF in these groups. We also evaluated the association between PEDF features and clinical, hormonal, and metabolic variables in the PCOS and control groups.

## Methods

### Study participants

This case–control study was carried out with women of reproductive age seen at the Gynecological Endocrinology Unit at Hospital de Clínicas de Porto Alegre, Brazil. Women with a body mass index (BMI) ranging from 18.5 to 39.9 kg/m^2^ were selected for the study. Twenty-two hirsute oligo-amenorrheic women (<9 cycles/year), presenting with increased levels of serum testosterone (>0.8 ng/mL) and/or free androgen index (FAI) (>6.1) and/or polycystic ovaries in the absence of other disorders causing hirsutism [21,22], were included. A control group was set up with 14 non-hirsute ovulatory women (regular cycles and luteal phase progesterone levels higher than 3.8 ng/mL) with normal androgen levels and normal ovaries on ultrasound, recruited through public advertisement at the same Gynecological Endocrinology Unit. None of the women from either group had received any drugs known to interfere with hormone levels, blood pressure, or metabolic variables for at least 3 months before the study. Women with diabetes, liver or kidney disease, thyroid dysfunction or pregnancy were excluded.

Twenty-two additional volunteers without laparoscopic evidence of pelvic disease were recruited for collection of subcutaneous and omental adipose tissue samples. This was done to ensure that PEDF gene expression could be reliably measured in scAT. The study protocol was approved by the Research and PostGraduate Group of Hospital de Clinicas de Porto Alegre (GPPG/HCPA) and the local Ethics Committee, and written informed consent was obtained from all participants.

### Study protocol

Medical interview and physical examination were performed as previously described [23,24]. Hirsutism was defined as a modified Ferriman-Gallwey score of 8 or more [25]. Blood pressure (BP) was measured in the sitting position after a 10-minute rest [26]. Anthropometric measurements included body weight, height, body mass index (BMI), waist circumference and waist/hip ratio (WHR) [23,27,28]. Hormonal and metabolic assessment as well as abdominal/transvaginal ultrasound were performed in all PCOS and control participants between days 2 and 10 of the menstrual cycle or on any day if the patient was amenorrheic. Polycystic ovary appearance (PCO) was defined as previously reported [5,29]. After an overnight 12-hour fast, blood samples were drawn from an antecubital vein between 8 a.m. and 10 a.m. for determination of serum PEDF and lipid profile at baseline, and glucose and insulin before and 2 hours after ingestion of 75 g oral anhydrous glucose (oral glucose tolerance test). Blood samples were also assessed for measurement of luteinizing hormone (LH), sex hormone–binding globulin (SHBG) and total testosterone (TT). Free androgen index was estimated by dividing TT (nmol/L) by SHBG (nmol/L) × 100. Homeostasis model assessment index to estimate insulin resistance (HOMA-IR) was calculated by multiplying insulin (mIU/mL) by glucose (mmol/L) and dividing this product by 22.5 [30]. Low-density lipoprotein (LDL) cholesterol was estimated indirectly with the Friedewald formula [31].

### Biochemical and hormonal assays

Total cholesterol, high-density lipoprotein (HDL) cholesterol, triglycerides, and glucose were determined by colorimetric-enzymatic methods (Siemens Advia System, Deerfield, IL, USA). Enzyme-linked immunosorbent assay (Chemicon International, Temecula, CA, USA) was used to measure total serum PEDF, with a sensitivity (S) of 0.9 ng/mL, intra-assay coefficient of variation (CV) of <5.3% and interassay CV <16%. As recommended by the manufacturer, to prevent PEDF from associating with other circulating proteins that may interfere with quantification of total serum concentration of this adipokine, samples were pre-treated with urea (8 M final concentration) for 60 minutes in ice and diluted 450 times in dilution buffer before being applied in duplicate to ELISA plates. Serum insulin (S = 0.2 mIU/mL), LH (S = 0.1 mIU/mL) and SHBG (S = 0.35 nmol/L) levels were measured with electrochemiluminescent immunoassays (Roche Diagnostics, Mannhein, Germany), with intra-assay CV <3% and interassay CV <5%. Total serum TT levels were measured with radioimmunoassay (Diagnostics Systems Laboratories Inc., Webster, TX), with S <0.1 ng/mL and CV <9.6%.

### Tissue collection

scAT biopsy was performed in all participants (22 PCOS and 14 control women) to obtain a 250 mg adipose tissue sample from the periumbilical region. To assess scAT and omental adipose tissue (omAT), another 22 normal women were recruited and samples were obtained through laparoscopy. Procedures were performed between days 2 and 10 of the menstrual cycle, or on any day if the patient was amenorrheic, by surgeons from the Plastic Surgery Service at Hospital de Clínicas de Porto Alegre or at the Human Reproductive Unit of Hospital Fêmina. Adipose tissue fragments were immediately frozen in liquid nitrogen and stored at −80°C until total RNA isolation.

### RNA isolation

Adipose tissue total RNA extraction was carried out in phenol-guanidine isothiocyanate (Trizol^®^, Invitrogen™ Life Technologies, Foster City, CA, USA) as previously described [32,33]. Concentration and quality of total RNA were assessed using a GeneQuant spectro-photometer (Pharmacia Biotech, Cambridge, England).

### Real-time RT-PCR protocol

Reverse transcription of 1 μg of total RNA into cDNA was carried out using the Superscript II First-Strand Synthesis System for RT-PCR (Invitrogen™ Life Technologies, Foster City, CA, USA), according to manufacturer instructions, in a PCT-100™ Programmable Thermal Controller (MJ Research Inc., Watertown, MA, USA). Real-time PCR was performed in triplicate in a 7500 Fast Real-Time PCR System thermal cycler with 7500 Fast System Sequence Detection 1.4 Software (Applied Biosystems, Foster City, CA, USA). SYBR^®^ Green dye fluorescence was detected by real-time monitoring experiments [34-36]. Primers were designed using the Primer Express 3.0 Software for Real-Time PCR (Applied Biosystems, Foster City, CA, USA), and acquired from Invitrogen™ Life Technologies (Foster City, CA, USA). Primer sequences were designed to target two exons of mRNA with respect to known splice variants and single-nucleotide polymorphism positions. The forward and reverse primer sequences designed for pigment epithelium-derived factor (NM_002615.5) were (5′ to 3′) CATCATTCACCGGGCTCTCTAC and GGCCTGGTCCCATATGACTTTT, respectively. These primers anneal between residues 463 to 484 (forward) and 654 to 633 (reverse), producing a 192-bp amplicon. mRNA quantitation was normalized to glyceraldehyde-3-phosphate dehydrogenase (NM_002046.3). The forward and reverse GAPDH primer sequences, ACCCACTCCTCCACCTTTG and CTCTTGTGCTCTTGCTGGG (5′ to 3′), respectively, anneal between residues 970 to 988 and 1,147 to 1,129, resulting in an amplicon of 178 bp. Complementary DNA samples (0.87 ng/μL) were mixed with a predetermined forward and reverse primer volume (0.5 and 0.7 μL for PEDF; 0.9 and 0.9 μL for GAPDH) and 12.5 μL of 2X Fast SYBR^®^ Green Master Mix (Applied Biosystems, Foster City, CA, USA) for a total volume of 25 μL. Protocol conditions consisted of denaturation at 94°C for 2 min followed by 50 cycles (30 sec, 94°C and 30 sec, 60°C). Amplicons produced single sharp peaks during melting curve analysis.

Data were analyzed by relative quantitation using the comparative C_T_ method [37]. Validation assays were performed by amplification of the target and reference genes, separately, using serial dilutions of an mRNA sample. Both target and reference mRNAs presented equal efficiencies of amplification. The ΔΔC_T_ method calculates changes in gene expression as relative fold difference between an experimental and calibrator sample, correcting for nonideal amplification efficiencies [38].

### Statistical analysis

Data were described as mean ± standard deviation (SD) or median [interquartile range]. The Wilcoxon two-related-samples test or the unpaired two-tailed Student’s *t*-test was used to compare group means for data with Gaussian distribution. The Mann–Whitney *U* test was used to compare group medians for data with non-Gaussian distribution. Pearson’s or Spearman’s rank correlation coefficients were calculated between variables using a two-tailed test for significance. A forward stepwise multiple regression model was also calculated using serum PEDF as a dependent variable, and fasting insulin and TT as independent variables. Log_10_ transformation was used to normalize the distribution of non-Gaussian variables, and these variables were back-transformed into their original units for presentation. Data were considered statistically significant at p < 0.05. The Statistical Package for the Social Sciences v. 18 (SPSS, Chicago, IL) was used for all statistical analyses.

## Results

**Figure 1:**
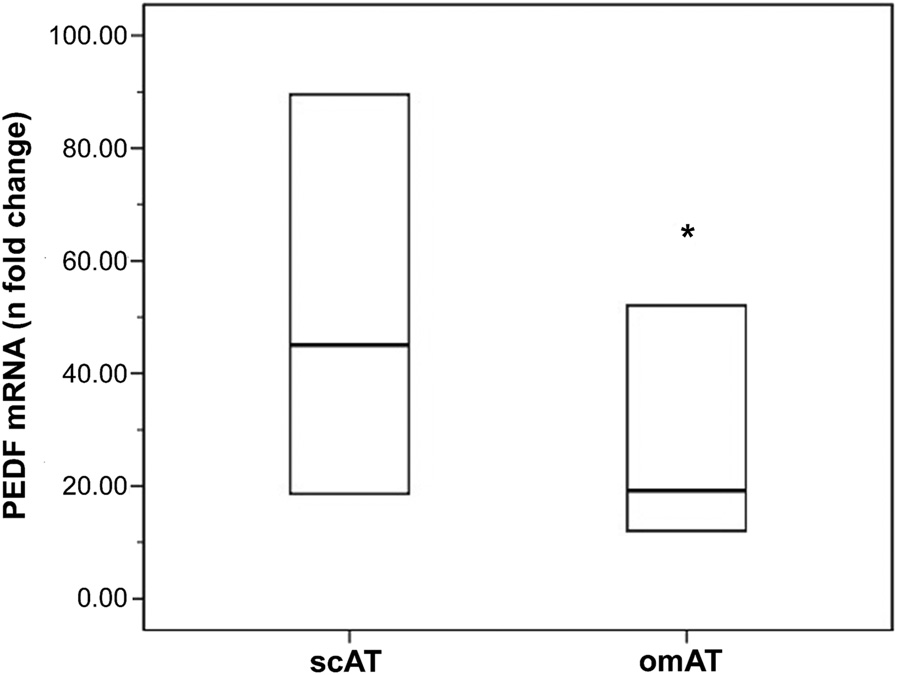
**PEDF gene expression in subcutaneous (scAT) and omental (omAT) adipose tissue of normal women (n = 22).** Messenger ribonucleic acid (mRNA) was expressed as *n*-fold-change difference from the calibrator sample (ΔΔC_T_ method). Values are expressed as median and interquartile range (lower and upper limit of the box). p = 0.042 in relation to scAT by Wilcoxon two-related-samples test.

Figure 1 shows PEDF gene expression in scAT and omAT from healthy controls (mean age, 33.3 ± 6.4 years). PEDF mRNA was significantly higher in scAT *versus* omAT (45.03 [18.57-94.06] and 19.17 [12.02-52.80] *n*-fold change in relation to calibrator sample, p = 0.042).

Most participants were white (86%). The remaining women were of mixed African and European ancestry. Concerning clinical presentation, 32% of PCOS patients were hypertensive and 9% had the metabolic syndrome. None of the controls had hypertension or metabolic syndrome. Age was similar in the PCOS and control groups (23.9 ± 6.2 *vs.* 25.4 ± 2.4 years, p = 0.501). Table 1 shows the clinical and hormonal features of women in the control and PCOS groups. BMI, waist circumference, WHR, BP, fasting glucose, HOMA-IR, triglycerides, HDL-cholesterol and SHBG were similar between the groups. As expected, hirsutism score, LH and TT were higher in PCOS (p ≤ 0.02). In addition, PCOS patients presented a worse metabolic profile, as indicated by higher 2 h glucose, total and LDL-cholesterol, and fasting and 2 h insulin levels than controls (p < 0.05). Between-group differences remained significant after adjustment for BMI, except for fasting and 2 h insulin.

**Table 1:** Clinical and hormonal features of women with PCOS and controls

**Characteristic**	**Control (n = 14)**	**PCOS (n = 22)**	**p**	p*
*Age (years)*	25.4 ± 2.4	23.9 ± 6.2	0.334	0.501
*BMI (kg/m*^*2*^*)*	26.9 ± 5.5	30.8 ± 5.9	0.058	
*Waist circumference (cm)*	85.5 ± 9.8	90.9 ± 13.7	0.321	0.166
*WHR*	0.78 ± 0.06	0.83 ± 0.09	0.120	0.079
*Ferriman-Gallwey score*	1 (0–3)	9 (8–14)	**0.003**	**0.003**
*Systolic BP (mmHg)*	111 ± 4	117 ± 16	0.096	0.291
*Diastolic BP (mmHg)*	68 ± 6	75 ± 12	0.178	0.187
*Glucose, fasting (mg/dL)*	87 ± 6	88 ± 6	0.555	0.597
*Glucose, 2 h (mg/dL)*	89 ± 13	117 ± 25	**0.007**	**0.006**
*Insulin, fasting (μIU/mL)*	7.8 (4.3-11.2)	12 (8–19)	**0.012**	0.089
*Insulin, 2 h (μIU/mL)*	56.6 (36.8-94.7)	101 (46–175)	**0.049**	0.110
*HOMA-IR*	1.8 (1.3-2.8)	2.6 (1.8-4.2)	0.083	0.100
*Triglycerides (mg/dL)*	54 (40–72)	77 (48–119)	0.135	0.441
*Total cholesterol (mg/dL)*	144 ± 35	178 ± 40	**0.013**	**0.026**
*HDL cholesterol (mg/dL)*	48 ± 15	53 ± 9	0.272	0.277
*LDL cholesterol (mg/dL)*	83 ± 27	109 ± 35	**0.024**	**0.036**
*LH (mIU/mL)*	6.0 (4.0-7.6)	10.9 (6.5-18.6)	**0.011**	**0.020**
*SHBG (nmol/L)*	44.9 (35.3-59.3)	34.9 (18.4-47.8)	0.173	0.360
*Total T (ng/mL)*	0.60 (0.47-0.72)	1.26 (0.91-1.43)	**<0.001**	**0.001**
*FAI*	4.7 (3.8-6.1)	9.4 (6.8-21.3)	**0.001**	0.101

Values are expressed as mean ± SD or median (interquartile range). Statistical differences by unpaired two-tailed Student’s *t*-test or Mann–Whitney *U* test. *BMI* body mass index, *BP* blood pressure, *FAI* free androgen index, *HDL* high-density lipoprotein, *HOMA-IR* homeostasis model assessment, *LDL* low-density lipoprotein, *LH* luteinizing hormone, *PCOS* polycystic ovary syndrome, *SHBG* sex hormone-binding globulin, *T* testosterone, *WHR* waist to hip ratio. *Adjusted for BMI (linear regression).

**Figure 2:**
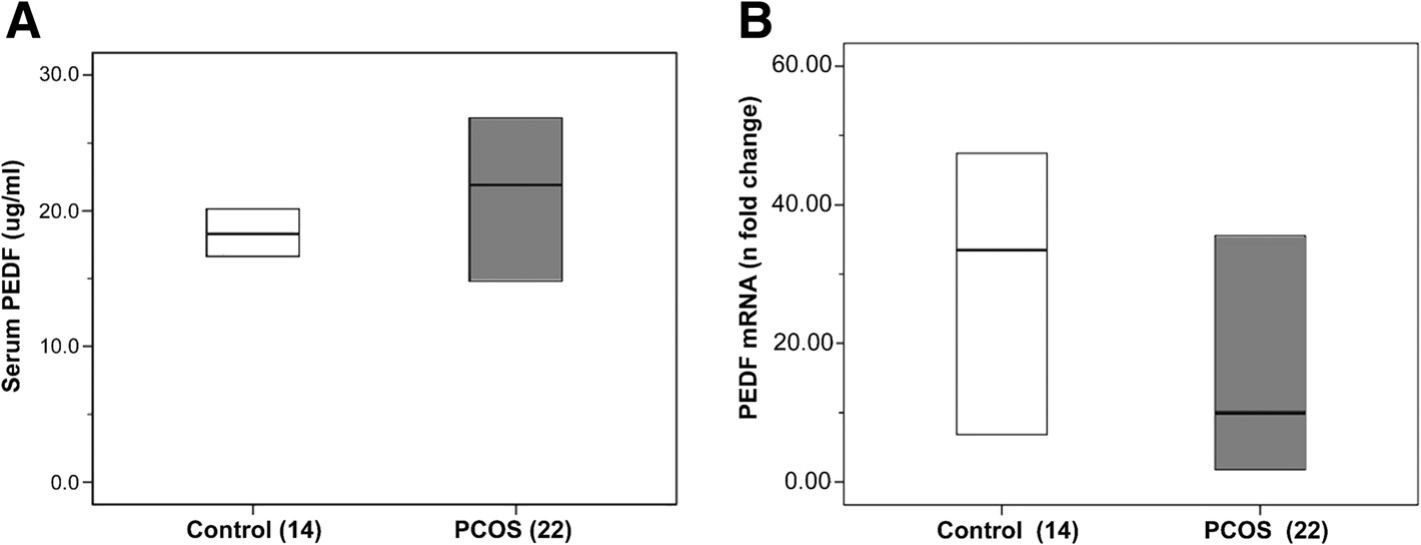
**Serum PEDF levels and PEDF gene expression. A)** Serum PEDF levels and **B)** PEDF gene expression in subcutaneous adipose tissue (scAT) in women with PCOS and controls. The number of biopsies analyzed per group appears within parentheses. Values are expressed as median and interquartile range (lower and upper limit of the box). PCOS: polycystic ovary syndrome. No significant between-group differences in PEDF serum levels (Student’s *t*-test). Messenger RNA was expressed as *n*-fold-change difference from the calibrator sample (ΔΔC_T_ method). No significant between-group differences in scAT PEDF gene expression (Mann–Whitney *U* test). Analyses were adjusted for BMI (linear regression).

Mean serum PEDF levels were similar in PCOS and control women (23.6 ± 11.0 *vs.* 18.6 ± 7.7 μg/mL, p = 0.622) (Figure 2A). Median PEDF scAT gene expression did not differ significantly between PCOS and controls (9.97 [1.52-41.40] *vs.* 33.46 [6.37-58.00] *n*-fold change in relation to calibrator sample, p = 0.509) (Figure 2B). No correlation was found between circulating PEDF levels and scAT PEDF gene expression (r = 0.116, p = 0.514). Serum PEDF correlated positively with BMI and waist circumference in both the PCOS and control groups (r = 0.556 and r = 0.506, respectively, p ≤ 0.004). In women with PCOS, serum PEDF also correlated with WHR, triglycerides, fasting insulin and HOMA-IR (r = 0.537, r = 0.473, r = 0.536 and r = 0.533, respectively, p ≤ 0.026). Multiple linear regression analysis revealed that insulin contributed positively and significantly to serum PEDF, independently of total testosterone, only in the PCOS group (Tables 2 and 3). Concerning the components of the metabolic syndrome, waist circumference greater than 88 cm was a significant independent predictor of serum PEDF in this study group, explaining 35% (p = 0.001) of variance in plasma PEDF concentrations.

**Table 2:** **Model-fitting results of stepwise regression for serum PEDF *vs. *significantly correlated variables for control women**

**Serum PEDF (ug/ml) *vs. *independent variables**	**Coefficient ± SE**	**β**	**p**
*Fasting insulin*	6.853 ± 8.731	0.236	0.451
*Total testosterone*	9.346 ± 13.197	0.213	0.495

r^2^ = 0.114.

*β* standardized regression coefficients. Values for fasting insulin and total testosterone were log_10_-transformed.

**Table 3:** **Model-fitting results of stepwise regression for serum PEDF *vs. *significantly correlated variables for PCOS women**

**Serum PEDF (ug/ml) *vs. *independent variables**	**Coefficient ± SE**	**β**	**p**
*Fasting insulin*	22.662 ± 9.379	0.565	**0.027**
*Total testosterone*	−4.590 ± 13.509	−0.080	0.738

r^2^ = 0.279.

*β* standardized regression coefficients. Values for fasting insulin and total testosterone were log_10_-transformed.

## Discussion

In the present study, serum PEDF levels and scAT gene expression did not differ between women with PCOS and age- and BMI-matched controls. The decision to measure PEDF gene expression in scAT was based on the finding that PEDF expression was higher in scAT than in omAT, as recently reported [39]. An advantage of using subcutaneous fat is that sample collection is less invasive than for omental fat.

One recent study reported that PEDF was related to obesity, but not to subcutaneous adiposity or insulin resistance, in obese women with PCOS [20]. On the one hand, this supports our hypothesis that PEDF does not originate from scAT. We observed that gene expression and circulating levels of scAT were not correlated in either PCOS or control women. No other significant correlations were found between subcutaneous PEDF expression and clinical, hormonal or metabolic variables, indicating that scAT production of PEDF is not the main determinant of serum PEDF concentrations. Moreno-Navarrete et al. [39] have demonstrated that liver, but not adipose tissue, might be the source of increased circulating PEDF, associated with insulin resistance, as previously proposed by other authors [16,17].

On the other hand, the findings of Joham et al. [20] indicate that PEDF does not play a causal role in the development of insulin resistance, strengthening the notion that women with PCOS have an intrinsic insulin-related dysfunction that might be exacerbated by, but not attributed to, obesity, as previously thought [40]. In our PCOS group, circulating PEDF levels were also associated with a worse metabolic profile as shown by positive correlations with triglycerides, fasting insulin and HOMA-IR. Data regarding the regulation of PEDF in women with PCOS, as well as in general populations are lacking. Recent work has implicated PEDF in the inducement of lipolysis, promoting lipid accumulation in muscle and liver and reducing fatty acid oxidation that was associated with insulin resistance [14]. In the general non-diabetic population, circulating PEDF has been related to aspects of the metabolic syndrome, including obesity, triglyceride levels, diastolic blood pressure, glucose, and insulin levels [12,17,41]. A few studies in patients with type 2 diabetes have demonstrated that PEDF levels were not only associated with the components of the metabolic syndrome, but also higher in proportion to the accumulation of the number of the components [13,16]. Few data are available in the literature concerning PEDF gene expression and metabolic syndrome or their isolated components. However, Nakamura et al. [16] showed that PEDF mRNA levels in cultured adipocytes were increased in parallel to the BMI values of subjects from whom adipocytes were derived, especially in omental adipocytes. In addition, Moreno-Navarrete et al. [39] reported that scAT PEDF gene expression was decreased in subjects with type 2 diabetes and did not change significantly after weight loss.

Conversely, a previous study has shown that PEDF serum levels were elevated in association with insulin resistance in normal-weight PCOS women when compared to BMI-matched controls [19]. While in that study, as in ours, serum PEDF levels were correlated with BMI in PCOS patients—suggesting that obese women with PCOS are also likely to have increased levels of PEDF—no significant elevation of PEDF was found in overweight/obese women with PCOS. However, in the study by Yang et al., only five women with PCOS were obese; therefore, the findings in these women were not conclusive. In the present study, the comparison between PCOS and control women covering a wide range of BMIs showed similar BMI-adjusted PEDF serum levels.

It is also interesting to note that, while there is evidence that circulating PEDF levels are higher in men than in women [12,17,41,42], and despite the higher TT found in our patients with PCOS as compared with control women, the testosterone-independent association between PEDF and insulin levels observed herein suggests that androgen excess does not play an essential role in PEDF circulating levels.

Limitations of the present study were the relatively small sample size, which precluded stratification of groups by BMI for comparison, and the lack of assessment of PEDF levels on different days of the menstrual cycle in control women. Therefore, further studies are required to investigate the associations between PEDF and clinical phenotype and pro-inflammatory markers in normal-weight *versus* obese women with PCOS, as well as to better characterize PEDF secretion over the course of the menstrual cycle.

## Conclusions

Serum levels and scAT gene expression of PEDF were similar in women with PCOS and age- and BMI-matched healthy controls, and were associated with metabolic risk factors. Furthermore, circulating levels of PEDF were not associated with subcutaneous adipose tissue gene expression in the study subjects, suggesting the existence of other sources for PEDF.

## Abbreviations

BMI: Body mass index; BP: Blood pressure; CV: Coefficient of variation; FAI: Free androgen index; HOMA: Homeostasis model assessment; LH: Luteinizing hormone; omAT: Omental adipose tissue; PCOS: Polycystic ovary syndrome; PEDF: Pigment epithelium-derived factor; scAT: Subcutaneous adipose tissue; SHBG: Sex hormone–binding globulin; TT: Total testosterone; WHR: Waist/hip ratio.

## Competing interests

The authors declare that they have no competing interests.

## Authors’ contributions

SBL, DMM and PMS were involved in the conception and design of the study, data collection and analysis. SBL and PMS drafted the article. All the authors read and approved the final manuscript.
